# Selection of the optimal trading model for stock investment in different industries

**DOI:** 10.1371/journal.pone.0212137

**Published:** 2019-02-13

**Authors:** Dongdong Lv, Zhenhua Huang, Meizi Li, Yang Xiang

**Affiliations:** 1 College of Electronics and Information Engineering, Tongji University, Shanghai, China; 2 School of Computer Science, South China Normal University, Guangzhou, China; 3 College of Information, Mechanical and Electrical Engineering, Shanghai Normal University, Shanghai, China; Shandong University of Science and Technology, CHINA

## Abstract

In general, the stock prices of the same industry have a similar trend, but those of different industries do not. When investing in stocks of different industries, one should select the optimal model from lots of trading models for each industry because any model may not be suitable for capturing the stock trends of all industries. However, the study has not been carried out at present. In this paper, firstly we select 424 S&P 500 index component stocks (SPICS) and 185 CSI 300 index component stocks (CSICS) as the research objects from 2010 to 2017, divide them into 9 industries such as finance and energy respectively. Secondly, we apply 12 widely used machine learning algorithms to generate stock trading signals in different industries and execute the back-testing based on the trading signals. Thirdly, we use a non-parametric statistical test to evaluate whether there are significant differences among the trading performance evaluation indicators (PEI) of different models in the same industry. Finally, we propose a series of rules to select the optimal models for stock investment of every industry. The analytical results on SPICS and CSICS show that we can find the optimal trading models for each industry based on the statistical tests and the rules. Most importantly, the PEI of the best algorithms can be significantly better than that of the benchmark index and “Buy and Hold” strategy. Therefore, the algorithms can be used for making profits from industry stock trading.

## Introduction

In the field of investment management, different types of asset allocation are one of the most important concerns of ordinary investors and portfolio managers. In terms of stock assets, it is a common practice to invest according to sectors or industries. For example, large fund companies often choose stocks of currently hot and growing industries, such as the high-tech sector and the cyclical consumer industry. Generally, due to industry policy orientation, economic cycles, industrial shift, and investor preferences, the stocks in the same industry have a similar trend and the trends of the stocks in different industries are often different. For example, we often choose stocks in the same industry (such as “MSFT” and “GOOG”, where they are the high-tech industries) as the object of pairs trading, and we can make a profit from their small price deviations. Meanwhile, we often choose stocks from different industries (such as “APA” and “DAL”, where “APA” is energy industry and “DAL” is consumer cyclical industry) to construct portfolios to disperse risk, which makes use of the weak correlation between their stock prices. Therefore, it is inappropriate to apply the same model to the stocks of all industries. In recent years, machine learning algorithms have made many exciting advances in stock quantitative trading. Researchers use support vector machines, decision trees, and other traditional machine learning algorithms to predict the future rise and fall of stock prices; they apply deep neural network technology to analyze sentiment of stock news texts to predict future price trends; they use adaptive reinforcement learning techniques for dynamic portfolio construction and market timing trading; they use online learning algorithm for optimal execution in the limit order book of a financial asset, and so on.

There are many machine learning algorithms for classification, including 1) the algorithms based on tree such as decision tree, random forest; 2) the algorithms based on distance such as support vector machine and K Nearest Neighbor (KNN); 3) the algorithms based on probability such as Naïve Bayes and logistic regression; 4) the algorithms based on a neural network such as multi-layer perceptron, recurrent neural network. These machine learning methods have their own merits and demerits, and they can be used to process different types of data sets. In our task, we model the rise and fall of stock prices in different industries, i.e., as a classification problem. We use the classification results of different algorithms as trading signals and formulate trading strategies based on the signals. Then, we conduct back-testing of these strategies and evaluate the performance of these classification models. If the trading performance of a model is statistically significantly better than that of other models in the same industry stock data set, we regard the model as the best trading model. In this way, we can complete the selection of the optimal trading models. However, as far as we know, there is no study from this perspective. Here, we put forward the question: are there statistically significant differences between the stock trading performance of different models in the same industry? That is, whether the performances of different algorithms significantly depend on industries or sectors? The problem constitutes the main motivation for this research, which is very important for quantitative investment practitioners and portfolio managers.

In this paper, we implement experiments on the SPICS and the CSICS, because they are the most active investment targets of the top two economies in the world today. We divide the two data sets into 9 industries respectively. For the stocks in each industry, we construct 44 technical indicators as shown in the appendix, including the KDJ index, cash flow index and so on. The label on the *T*-th trading day is the sign for the yield of the *T+1*-th trading day relative to the *T*-th trading day. That is, if the yield is positive, the label value is set to 1; otherwise, it will be set to 0. For each stock, we choose the technical indicators of 2000 trading days before December 31, 2017, to build a stock dataset. After the dataset of a stock is built, we choose the walk-forward analysis method to train the machine learning models on several rounds. In each round of training, we train traditional machine learning methods such as support vector machine (SVM), random forest (RF), logistic regression (LR), naïve Bayes model (NB), classification and regression tree (CART), eXtreme Gradient Boosting algorithm (XGB) and deep neural network models such as Multilayer Perceptron (MLP), Deep Belief Network (DBN), Stacked Auto-Encoders(SAE), Recurrent Neural Network(RNN), Long Short-Term Memory(LSTM), Gated Recurrent Unit(GRU), and then forecast the trends of stock prices in different industries. Finally, we adopt the metrics, such as winning ratio (WR), annualized return rate (ARR), annualized Sharpe ratio (ASR) and maximum drawdown (MDD) to evaluate the trading performance of various methods and then select the optimal model for each industry based proposed a series of rules.

The experiment results show that we can select the optimal trading models for all industries based on sifting rules and refining rules; in most industries, the ARR and ASR of the optimal algorithms can be significantly better than that of benchmark index and BAH strategy; the MDD of the best algorithms can be significantly lower than that of BAH strategy. Therefore, the algorithms can be applied to risk management and automated stock trading in different industries.

The remainder of this paper is organized as follows: Section 2 reviews the stock forecasting models in the existing literature including the methods of traditional machine learning and the methods based on the deep neural network. Section 3 describes the method of data preparation. Section 4 gives the parameter settings of all machine learning algorithms and the trading signal generating algorithm of the models mentioned in this paper. Section 5 gives the performance evaluation indicators for back-testing, and evaluates the performance of the algorithm in the different industries and select the optimal models for each industry. Section 6 provides a comprehensive conclusion and future research directions.

## Literature review

Predicting the future price trends of stock and making investment decisions are very big challenge. Nevertheless, academic researchers and industry practitioners are trying to adopt more suitable theories and methods to implement stock trading and expect to make profits.

### Traditional machine learning models

Traditional machine learning models map the feature space to the target space. The parameters of the learning model are less. Therefore, the learning goal can be better accomplished in the case of fewer data. Moreover, traditional machine learning algorithms usually use interpretable mathematical methods such as support vector machines to build a learning task or model learning tasks based on clear and explicit rules such as decision trees. Huang et al. used SVM to forecast the weekly movement direction of the NIKKEI 225 index and compared its performance with Linear Discriminant Analysis [1]. Chen applied SVM to do pattern recognition in the financial engineering domain [2]. Xie used SVM to forecast the closing price on the third day and optimized the parameters of the model with particle swarm algorithm [3]. Ladyzynski et al. presented a novel architecture of the system for automated stock trading, which applied RF, trend detection tests and force index volume indicators to investigate if machine learning was able to predict future trends. The results showed that the system failed to generate a profitable trading strategy [4]. Zhang et al. used an unsupervised heuristic algorithm to cut transaction data into four main classes, and the class prediction models were trained by a combination of RF, imbalance learning and feature selection [5]. Ruta used LR as the class method and learned to generate profit from multiple inter-market price predictions and markets’ correlation [6]. Patel compared four stocks predicted models, ANN, SVM, RF, and NB on 10 years of two group historical data, and the results showed that using trends deterministic data could improve predicted performance [7]. Luo et al. integrated piecewise linear representation (PLR) and weighted SVM to forecast the stock trading signals, and the comparative experiments on 20 shares from Shanghai Stock Exchange in China showed that the predicted accuracy and profitability was effective [8]. Zbikowski used volume weighted SVM with walk-forward testing and feature selection for the purpose of creating a stock trading strategy, and the trading strategy results of given methods could improve trading performance [9]. Dash et al. proposed a novel decision support system using a computational efficient functional links artificial neural network and a set of rules to generate the trading decision [10].

### Deep neural network models

In recent years, the applications of deep neural network algorithms in finance have attracted more and more attention. These algorithms mainly connect some neurons into multiple layers to form a complex deep neural network structure. Through this complex structure, the mapping relationship between input and output is established. As the number of layers of the neural network increases, the neural network can automatically adjust the weight parameters to extract advanced features. The deep neural network models have many parameters compared with the traditional machine learning models, so the performances of deep neural network models tend to increase as the amount of data grows. Of course, deep learning has high requirements for computing hardware; deep neural networks use nested hierarchy structure to perform representation learning, so deep learning algorithms are less interpretable. Bao et al. presented a deep learning framework, which combined wavelet transform(WT), SAE and LSTM for stock price forecasting [11]. Thomas et al. deployed LSTM to predicted out-of-sample directional movements for the constituent stocks of the S&P 500 index [12]. Makickiene et al. proposed a new method of orthogonal input data to improve the process of RNN learning and financial forecasting [13]. Persio et al. compared different RNNs architectures such as multi-layer RNN, LSTM and GRU performances on forecasting Google stock price movements [14]. Dunis et al. applied three different types of neural network including MLP and RNN to trade oil futures spreads in the context of a portfolio of contracts [15]. Chong et al. proposed a systematic analysis of the use of deep learning networks for stock market analysis and prediction, and examine the effect of three unsupervised feature extraction methods on the ability of deep neural networks to forecast future market behavior [16]. Krauss et al. implemented and analyzed the effectiveness of deep neural networks, gradient-boosted-trees, RF, and several ensembles of these methods in the context of statistical arbitrage, and the experimental findings were promising [17]. Hsieh et al. used WT and RNN to forecast stock markets, which based on an artificial bee colony algorithm [18]. Längkvist et al. gave a review of some development in deep learning and unsupervised learning for time series problems and pointed out some challenges in this area [19]. Liu et al. gave some widely-used deep learning architectures and their applications, and the models included autoencoder, DBN, and restricted Boltzmann machine(RBM) [20]. Dixon applied RNNs to high- frequency trading and solved a short sequence classification problem of limit order book depths and market orders to predict the next event price-flip [21]. Kim et al. proposed a hybrid LSTM model to predict stock price volatility that combined the LSTM with various GARCH-type models [22]. Shen et al. applied GRU and its improved version for forecasting trading signals for three stock indexes and compared proposed models with the traditional deep network and the other popular models [23]. Sezer et al. proposed a deep neural network based stock trading systems evolutionary optimization technical analysis parameters to improve the stock trading performance [24].

## Data preparation

### Data acquisition

In this paper, we conduct experiments on SPICS in US and CSICS in China, which represent the stock markets of the most actively developed and emerging economies in the world. They have attracted many investors' attention and are one of the most important markets for global asset allocation. The reason for our choice of SPICS is that it contains a wide range of industries, including industrial stocks, high-tech stocks, public utility stocks, financial stocks and so on, which account for more than 80% of the total market value of the US stock. These stocks have strong liquidity and can provide a good object for the test of trading strategies. At the same time, the selection criteria of CSICS are scale and liquidity, and it accounts for more than 60% of the total market value of China's A-share listed companies. It is worth noting that both SPICS and CSICS are dynamically adjusted according to certain rules. Therefore, the stocks that do not meet the requirements in a certain period will be removed from the original samples. In the experiment, we select the data from the past 2000 trading days of SPICS and CSICS before December 31, 2017, respectively. Therefore, in order to get enough data for the experiments, we have removed the stocks that have been suspended, delisting and less than 2000 trading days. Finally, we select 424 SPICS and 185 CSICS, which account for about 85% and 60% of the total number of stocks respectively.

We grab the price data (the highest price, the lowest price, the opening price, the closing price) and the volume data of the SPICS from http://finance.yahoo.com and the data of the CSICS from http://quotes.money.163.com. The acquired data is not processed by ex-dividend/rights, so we need to process these data according to the dividend and rights issue announced by listed companies. Because rationed shares, increase shares by transferring, and dividends can cause excessive jump and distortion of stock price, which will affect the performance of trading algorithms and back-testing.

### Feature generation

In this paper, we select 44 relatively well-recognized technical indicators with a high frequency of use as the features, which include trend indicators, the volatility indicators, cash flow indicators, investor psychological indicators and so on, as shown in supporting information (S1 Table). These features describe the dynamic change of a stock price and volume in the trading day. It is worth noting that the number of technical indicators of stocks is large, and the same indicator can generate many different indicators because of the different parameters. In addition to some common indicators such as commodity channel index (CCI) and relative strength index (RSI), there are some other indicators such as average true range (ATR), triple exponentially smoothed moving average (TRIX), because these indicators are of great significance for characterizing the movement pattern of stocks.

### Data normalization

Data normalization is an important step in data preprocessing. Normalized data are generally used as inputs to machine learning and data mining models. The significance of Normalization is to compress all data to [0,1]. In this way, a larger value of features can be avoided having a strong influence on the output of the model, so as to improve the robustness of the model. In this article, we adopt max-min normalization. That is, to each feature *x*∈*R*^*n*^, we have x* = (*x*−min(*x*))/(max(*x*)−min(*x*)).

## Trading algorithm and its design

### Learning algorithm

Given a training dataset, *D* = {(*x*_1_,*y*_1_),(*x*_2_,*y*_2_),⋯,(*x*_*P*_,*y*_*P*_)}, where *x*_*i*_ = {*x*_*i*1_,*x*_*i*2_,⋯,*x*_*iP*_} is an instance of input; *P*is the number of sample features; *y*_*i*_ = {0,1} is a class label; *i* = 1,2,3,⋯,*N*, where *N* is the sample size. *D* is a matrix of N*(*P*+1), where the *P*+1-th column of *D* is class label. The task of learning is to construct a learning model based on a given training dataset so that the model can classify class labels correctly. In this paper, we will use the six traditional machine models, namely LR, SVM, CART, RF, BN, XGB and six deep neural networks, namely MLP [25], DBN [26], SAE [27], RNN [28], LSTM [28], and GRU [28] as classifiers to predict the rise and fall of the stock prices. The main model parameters and training parameters of these learning algorithms are shown in the above table.

In Table 1 and Table 2, features and class labels are set according to the input format of various machine learning algorithms in R language. *Matrix* (*m*, *n*) represents a matrix with m rows and n columns; *Array* (*p*, *m*, *n*) represents a tensor (namely array in R language), where each layer of the tensor is Matrix (m, n) and the height of the tensor is *p*. *c* (*h1*, *h2*, *h3*, …) represents a vector, where the length of the vector is the number of hidden layers and the *i*-th element of *c* is the number of neurons of the *i*-th layers. In the experiment, m = 250 represents that the data of the past 250 days (about 250 trading days in a year) are used as training samples in each round of walk-forward analysis, because we think the model trained with one year's data is enough to predict the day ahead; n = 44 represents that the data of each day has 44 features. In Table 2, the activation function of all deep neural network models is a sigmoid function. Other parameters such as learning rate, batch size, and epoch are all the default values in the algorithm of R programs.

**Table 1:** Main parameter setting of traditional machine learning algorithms.

	Features	Label	Main parameters
LR	*Matrix*(250,44)	*Matrix*(250,1)	A specification for the model link function is logit.
SVM	*Matrix*(250,44)	*Matrix*(250,1)	The kernel function used is Radial Basis kernel; Cost of constraints violation is 1.
CART	*Matrix(*250,44)	*Matrix*(250,1)	The maximum depth of any node of the final tree is 20; the splitting index can be Gini coefficient.
RF	*Matrix*(250,44)	*Matrix(*250,1)	The Number of trees is 500; Number of variables randomly sampled as candidates at each split is 7.
BN	*Matrix*(250,44)	*Matrix*(250,1)	the prior probabilities of class membership is the class proportions for the training set.
XGB	*Matrix*(250,44)	*Matrix*(250,1)	The maximum depth of a tree is 10; the max number of iterations is 15; the learning rate is 0.3.

**Table 2:** Parameter setting of deep neural network algorithms.

	Features	Label	Learning rate	Dimensions of hidden layers	Activation function	Batch size	Epoch
MLP	*Matrix*(250,44)	*Matrix*(250,1)	0.8	*c*(25,15,10,5)	sigmoid	100	3
DBN	*Matrix*(250,44)	*Matrix*(250,1)	0.8	*c*(25,15,10,5)	sigmoid	100	3
SAE	*Matrix*(250,44)	*Matrix*(250,1)	0.8	*c*(20,10,5)	sigmoid	100	3
RNN	*Array(*1,250,44)	*Array*(1,250,1)	0.01	*c*(10,5)	sigmoid	1	1
LSTM	*Array(*1,250,44)	*Array*(1,250,1)	0.01	*c*(10,5)	sigmoid	1	1
GRU	*Array*(1,250,44)	*Array*(1,250,1)	0.01	*c*(10,5)	sigmoid	1	1

It is worth noting that experimental data is high-dimensional time series data. Previous studies have shown that time series data have autocorrelation and time dependencies, so it is different from the assumption of independent and identically distributed data in machine learning model. Therefore, we do not divide the data set into training dataset, validation dataset and test dataset in the experiment, because the validation dataset can separate the training dataset and the test dataset, which will cause the dependency between time series to disappear. Meanwhile, time series data are not suitable for cross-validation to optimize parameters because it is logically wrong to use the data after a certain time to predict the data before that time. Therefore, we do not use validation dataset to choose hyper-parameters. The hyper-parameters mentioned in the paper such as the number of layers of the deep neural network and the number of neurons in each layer are empirically tuned based on previous experiments. For those insensitive parameters such as the number of trees in the random forest algorithm and the prior probabilities of class membership in naïve Bayesian algorithm, we use the default parameters preset by R packages.

### Walk-forward analysis

**Fig 1:**
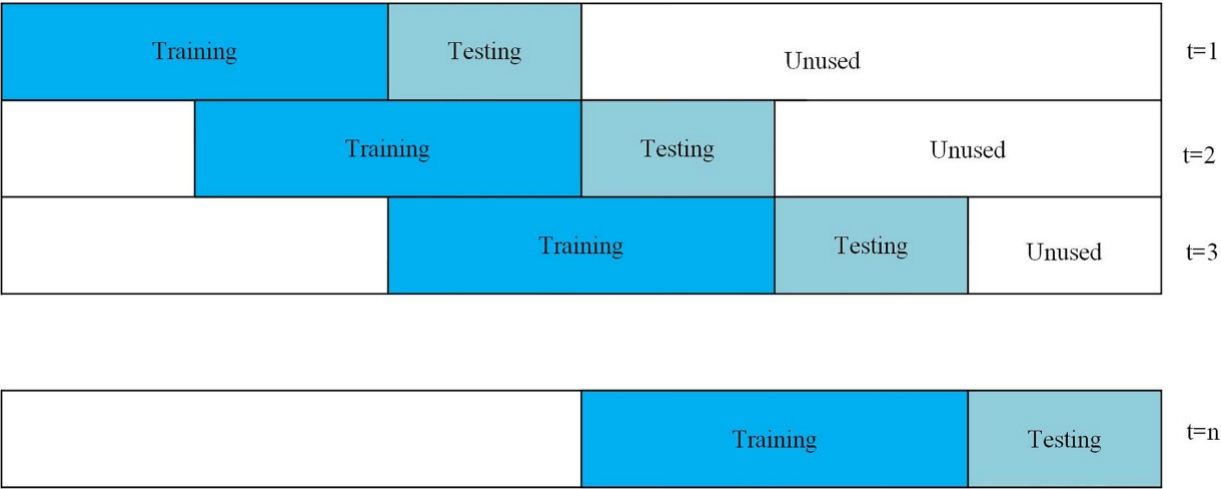
The schematic diagram of walk-forward analysis.

Walk-Forward Analysis [29] is a systematic and formalized manner of performing what has been referred to as a rolling optimization or a periodic re-optimization (see Fig 1). One of the primary benefits of the walk-forward analysis is to determine the robustness of the trading strategy. Walk-forward analysis is to determine the degree of confidence with which the trader may anticipate that the strategy will perform in real-time trading.

Another important advantage of walk-forward analysis is to produce a better trading performance as markets, trends, and volatility change. Since this periodic re-optimization is done with a strategy-appropriate amount of current price data, this also provides an efficient way to continuously adapt a trading model to ongoing changes in market conditions.

### The algorithm for generating trading signals

In this part, we use machine learning algorithms as the classifiers to predict the ups and downs of the stocks in each industry of SPICS and CSICS and use the prediction results as the signals of daily trading. We use the walk-forward analysis method to train each machine learning algorithm step by step. In each step, we use the data from the past 250 days (one year) as the training set and the data for the next 5 days (one week) as the test set. Each stock contains data for 2,000 trading days, so it takes (2000–250) / 5 = 350 training sessions to produce a total of 1,750 predictions which are the signals of daily trading. The algorithm for generating trading signals is shown in Algorithm 1.

**Algorithm 1**. **Generating Trading Signals in R Language**

**Input**: Stock Code List for each industry (SCLEI)

**Output**: Trading Signals

    1. N = length of Stock Code List #424 SPICS, and 185 CSICS. N = 424, 185.

    2. L = Number of Samples #L = 2000

    3. P = Length of Features #P = 44

    4. k = length of Training Dataset #k = 250

    5. n = Length of Testing Dataset/Length of Walk-Forward Window #n = 5

    6. **for** i in 1:N

    7.    Stock Data = SCLEI[i]

    8.    M = (L-k)/n

    9.    Trading Signal0 = NULL

    10.    **for** j in 1:M

    11.        New_Data = Stock Data[(k+n*(j-1)):(k+n+n*(j-1)), ]

    12.        New_Train = New_Data[1:k,]

    13.        New_Test = New_Data[(k+1): (k+n),1:P]

    14.        Train_Model = Learning Algorithm(New_Train)

    15.        Proba = Train_Model(New_Test)

    16.        **if** Proba> = 0.5 **then**

    17.            Trading Signal0 = 1

    18.        **else**

    19.            Trading Signal0 = 0

    20.        **End if**

    21.        Trading Signal = c(Trading Signal, Trading Signal0)

    23.    **End for**

    24.    return (Trading Signals)

    25. **End for**

## Performance evaluation and optimal trading model selection

### Performance evaluation indicators

Investment performance is an important tool to evaluate the effectiveness of a quantitative trading algorithm. In this paper, we use 12 machine learning algorithms and walk-forward analysis to predict the future trends of stock prices. Then, we use the classification forecast results as the trading signal to conduct the back-testing and apply the WR, ARR, ASR, and MDD as the indicators of the trading performance evaluation. These indicators reflect the investment ability of investors or trading algorithms.

WR is the ratio of the number of days with positive earnings to the total number of the trading day. It is noteworthy that our trading strategies do not allow short selling. So, we cannot trade when our trading algorithms predict that the stock prices will fall. WR is a measure of the accuracy of trading signals, and a better algorithm for generating trading signals will lead to a higher WR. As the most basic evaluation indicator, WR can be used to assess whether the current transaction performance is consistent with the previous one. The decline in the WR may indicate that the trading strategy has reached saturation.

*ARR* is a theoretical rate of return, not the real yield of investment strategy. It is derived from the average rate of return in the past investment period by the annualized calculation process and is not representative of future performance. Suppose that the holding period of an investment tool is *H*, the return rate of the investment tool is *RR*_*H*_, and there are *m* single periods in one year. *ARR* is given by the following formula.

$$ARR=[{\left(1+RR_{H}\right)}^{1/H}{]}^{m}-1$$

This method takes into consideration the continuous compound interest of multiple periods. In some cases, *ARR* = m*R_*H*_/*H* can be used to calculate *ARR*. In general, we first calculate the yield of a single period and then calculate the *ARR*.

*ASR* is a performance evaluation index designed by Sharpe in 1966 [30]. It is a risk-adjusted return. Suppose that the holding period of an investment tool is *H*, and there are *m* single periods in a year. In the *H* period, the *ARR* of the investment tool is *ARR*_*H*_, the standard deviation of return rate is *σ*_*H*_, and *R*_*f*_ is the benchmark such as risk-free return. In this paper, we set *R*_*f*_ = 0. *ASR* is given as follows.

$$ASR=\sqrt{m}*\left(ARR_{H}-R_{f}\right)/\sigma_{H}$$

Drawdown is a measure of historical loss. It is the largest loss compared to the previous highest value (water level) of the net value curve. Investment managers usually get performance fees after their investment returns exceed the water level. *MDD* shows the largest decline in the price or value of the investment period *H*, which is an important risk assessment indicator. In the period of investment *τ*, we first calculate the *D*_*τ*_ at any time *τ*≤*H*. Then, we can get the *MDD*_*H*_ when we go traverse the whole interval.

$$D_{\tau }=\max\left(0,\underset{t\in \left(0,\tau \right)}{\max p_{t}}-p_{\tau }\right);MDD_{H}=\underset{\tau \in \left(0,H\right)}{\max D_{\tau }}/\underset{\tau \in \left(0,H\right)}{\max p_{\tau }}$$

where *p*_*t*_ denotes the value of the net value curve with time *t*; *D*_*τ*_ represents the drawdown at the time *τ*, i.e., the difference between the maximum value in [0,*τ*] and the value of at the time *τ*. *MDD*_*H*_ denotes the maximum drawdown in [0,*H*].

It is noteworthy that we do not consider transaction costs when calculating these performance evaluation indicators. Stocks may be traded only once in a few days when we implement stock daily trading strategy and short selling is not allowed. So, transaction costs are few and even negligible.

### Evaluation and analysis of trading performance for the two datasets

In order to study the significant difference among the application of different machine learning algorithms in different industries, we divide the industry into 9 categories based on finance.sina.com.cn, which including Basic Materials (BM), Consumer Cyclical (CC), Communication (COM), Energy (EN), Finance (FIN), industry (IND), Non-Consumer Cyclical (NCC), Public Utility (PU), and Technology (TECH) as shown in supporting information (S2 Table). The number of SPICS and CSICS in various industries is shown in Table 3.

**Table 3:** The number of two index component stocks in various industries.

	BM	CC	COM	EN	FIN	IND	NCC	PU	TECH	Total
CSICS	20	14	9	11	44	42	19	8	18	185
SPICS	18	57	32	35	75	59	82	26	40	424

In order to compare whether there are statistically significant differences between the stock trading performance of different algorithms in the same industry, we put forward the following test hypotheses:

For any industry *i*∈{*BM*,*CC*,*COM*,*EN*,*IND*,*NCC*,*PU*,*TECH*}, for any performance evaluation indicator *j*∈{*WR*,*ARR*,*ASR*,*MDD*}.The null hypothesis *a* is *Hija*, alternative hypotheses *b* is *Hijb*.

*Hija*: in the industry *i*, the evaluation indicator *j* of all trading strategies are the same;

*Hijb*: in the industry *i*, the evaluation indicator *j* of all trading strategies are not the same.

Given the significance level is 0.05. We apply two non-parametric statistical test method. Firstly, we use the Kruskal-Wallis rank sum test [31]to carry out the analysis of variance. Secondly, if the alternative hypothesis is established, we need to apply the Nemenyi test [32] to do the multiple comparisons between trading strategies. In this process, we use the index (S&P 500 index and CSI 300 index) and BAH strategy as the benchmark.

#### Comparative analysis of intelligent trading algorithms for each industry in SPICS

In this part, we will analyze whether there are statistically significant differences in the WR, ARR, ASR, and MDD among different algorithms for each industry in SPICS, which can provide guidance for using different trading algorithms in different industries.

From Table 4, we can see that in all industries, MLP achieves the highest WR among all algorithms. Through the multiple comparison analysis, we can find that the WR of MLP is not significantly different from that of DBN, SAE, RNN, GRU, LSTM, and SVM in BM, but the WRs of MLP, SAE, and DBN are significantly greater than that of other algorithms. In CC, FIN, IND, NCC, TECH, there is no significant difference between MLP, DBN, and SAE for WR, but the WRs of them is significantly greater than other algorithms. In COM, the WR of MLP is not significantly different from that of DBN and SAE, but the WR of MLP is significantly greater than that of other algorithms except for SAE and DBN; the WRs of DBN and SAE are not significantly different from that of LSTM, but the WRs of them is significantly greater than other algorithms. In EN, there is no significant difference between the WR of all trading algorithms. In PU, the WR of MLP is not significantly different from that of DBN that SAE, but the WRs of MLP, SAE, and DBN are significantly greater than other algorithms.

**Table 4:** Comparison of the WR of different trading algorithms in the different industries of SPICS. Best performance is in boldface.

	BM	CC	COM	EN	FIN	IND	NCC	PU	TECH
MLP	**0.7175**	**0.7851**	**0.7900**	**0.5412**	**0.8440**	**0.8078**	**0.8554**	**0.9013**	**0.8043**
DBN	0.7108	0.7754	0.7792	0.5312	0.8354	0.7994	0.8439	0.8955	0.7914
SAE	0.7112	0.7763	0.7826	0.5274	0.8374	0.7982	0.8484	0.8954	0.7967
RNN	0.5239	0.5221	0.5260	0.4996	0.5283	0.5187	0.5281	0.5385	0.5336
LSTM	0.5256	0.5283	0.5296	0.5252	0.5298	0.5298	0.5314	0.5333	0.5271
GRU	0.5218	0.5118	0.5110	0.5189	0.5082	0.5171	0.5081	0.5185	0.5143
CART	0.5024	0.5155	0.5155	0.4957	0.5188	0.5142	0.5138	0.5213	0.5194
NB	0.4881	0.4839	0.4724	0.5146	0.4800	0.4741	0.4763	0.4874	0.5001
RF	0.5052	0.5229	0.5225	0.4996	0.5189	0.5127	0.5168	0.5094	0.5203
LR	0.5106	0.5240	0.5106	0.4959	0.5112	0.5218	0.5213	0.5170	0.5255
SVM	0.5478	0.5606	0.5602	0.5068	0.5794	0.5747	0.5778	0.5809	0.5744
XGB	0.5071	0.5155	0.5191	0.5006	0.5169	0.5129	0.5142	0.5055	0.5171

From Table 5, we can see that the ARR of CART is the highest in the BM, COM, EN, and IND; the ARR of DBN is the highest in the CC and PU; the ARR of MLP is the highest in FIN and NCC; the ARR of SAE is the highest in TECH. Through the multiple comparison analysis, we can find that the ARR of S&P 500 index is not significantly different from that of BAH strategy, but the ARRs of all machine learning algorithms are significantly greater than that of S&P 500 index and BAH in the BM, CC, COM, EN, IND, PU, and TECH; otherwise, there is no significant difference between the ARRs of any two algorithms. In the FIN, the ARR of S&P 500 index is not significantly different from that of BAH strategy, but the ARRs of all machine learning algorithms are significantly greater than that of S&P 500index and BAH strategy; the ARR of MLP is significantly greater than that of LR; the ARRs of MLP, DBN, and SAE are significantly greater than that of LSTM; otherwise, there is no significant difference between the ARRs of any two algorithms. In NCC, the ARR of the S&P 500 index is significantly lower than that of BAH strategy; the ARRs of all machine learning algorithms are significantly greater than that of S&P 500 index and BAH strategy; otherwise, there is no significant difference between the ARRs of any two algorithms.

**Table 5:** Comparison of the ARR of various trading strategies in the different industries of SPICS. Best performance is in boldface.

	BM	CC	COM	EN	FIN	IND	NCC	PU	TECH
Index	0.1226	0.1225	0.1227	0.1229	0.1226	0.1228	0.1227	0.1228	0.1228
BAH	0.1461	0.1809	0.1598	0.0533	0.1642	0.1742	0.1743	0.1257	0.1975
MLP	0.3312	0.3837	0.3334	0.2713	**0.3690**	0.3283	**0.3121**	0.2410	0.3608
DBN	0.3304	**0.3838**	0.3254	0.2720	0.3658	0.3211	0.3077	**0.2455**	0.3517
SAE	0.3422	0.3863	0.3316	0.2731	0.3657	0.3246	0.3085	0.2418	**0.3634**
RNN	0.3239	0.3164	0.2962	0.2724	0.3251	0.2940	0.2707	0.2054	0.3177
LSTM	0.2889	0.3142	0.2937	0.2790	0.3192	0.2914	0.2712	0.2085	0.3197
GRU	0.2896	0.3071	0.2930	0.2749	0.3275	0.2937	0.2758	0.2060	0.3215
CART	**0.3533**	0.3638	**0.3508**	**0.3143**	0.3628	**0.3340**	0.2996	0.2190	0.3554
NB	0.3111	0.3198	0.3003	0.2615	0.3305	0.2982	0.2745	0.2171	0.3269
RF	0.3198	0.3454	0.3205	0.2560	0.3419	0.3170	0.2986	0.2240	0.3393
LR	0.3013	0.3213	0.2981	0.2773	0.3157	0.2935	0.2754	0.2133	0.3176
SVM	0.3124	0.3291	0.3181	0.2546	0.3336	0.3145	0.2931	0.2143	0.3359
XGB	0.3302	0.3246	0.3172	0.2552	0.3345	0.3079	0.2871	0.2121	0.3288

From Table 6, we can see that the ASR of XGB is the highest in the BM and COM; the ARR of SAE is the highest in the EN; the ARR of RF is the highest in the CC, FIN, IND, NCC, PU, and TECH. Through the multiple comparison analysis, we can find the ASR of S&P 500 index is not significantly different from that of BAH strategy, but the ASRs of all trading algorithms are significantly greater than that of S&P500 index and BAH strategy in all industries except EN. Otherwise, there is no significant difference between the ASRs of any two algorithms in the BM, COM, PU, and TECH. In the CC, the ASR of LR is significantly greater than that of CART; otherwise, there is no significant difference between the ASRs of any two algorithms. In the EN, the ASR of S&P 500 index is not significantly different from that of RNN, LSTM, GRU, CART, NB, RF, LR, SVM, and XGB, but the ASRs of BAH strategy, MLP, DBN, and SAE is significantly greater than that of S&P 500 index; the ASRs of all trading algorithms are significantly greater than that of BAH strategy; otherwise, there is no significant difference between the ASRs of any two algorithms. In the FIN, the ASRs of all trading algorithms are significantly greater than that of CART; otherwise, there is no significant difference between the ASRs of any two algorithms. In the IND, the ASR of RF is significantly greater than that of DBN; the ASR of CART is significantly lower than that of NB, RF, SVM, XGB; otherwise, there is no significant difference between the ASRs of any two algorithms. In the NCC, the ASR of RF is significantly greater than that of MLP, DBN, SAE; the ASR of CART is significantly lower than that of RF, SVM, XGB; otherwise, there is no significant difference between the ASRs of any two algorithms.

**Table 6:** Comparison of the ASR of various trading strategies in the different industries of SPICS. Best performance is in boldface.

	BM	CC	COM	EN	FIN	IND	NCC	PU	TECH
Index	0.8370	0.8363	0.8374	0.8388	0.8368	0.8382	0.8377	0.8382	0.8382
BAH	0.5779	0.6997	0.6060	0.1827	0.6620	0.7398	0.7492	0.7267	0.7039
MLP	1.5021	1.6770	1.4680	1.1954	1.7345	1.6042	1.4916	1.4771	1.4775
DBN	1.5153	1.6912	1.4418	1.1879	1.7281	1.5756	1.4880	1.5084	1.4604
SAE	1.5601	1.6982	1.4631	**1.2014**	1.7250	1.5974	1.4805	1.4880	1.5001
RNN	1.5684	1.6165	1.4893	1.0890	1.7561	1.6925	1.5763	1.5577	1.5273
LSTM	1.4218	1.5987	1.4766	1.0613	1.7282	1.6649	1.5712	1.5999	1.5243
GRU	1.4690	1.5672	1.4832	1.0752	1.7811	1.7021	1.6297	1.5739	1.5456
CART	1.4027	1.4497	1.3751	1.0308	1.4442	1.4879	1.4171	1.3933	1.3544
NB	1.6038	1.6652	1.5602	1.0113	1.7805	1.7413	1.6407	1.7193	1.5999
RF	1.6010	**1.7470**	1.6057	0.9991	**1.8090**	**1.8066**	**1.7420**	**1.7637**	**1.6307**
LR	1.4874	1.6519	1.5317	1.0781	1.7156	1.6955	1.5995	1.6484	1.5115
SVM	1.5543	1.6337	1.5675	1.0094	1.7376	1.7504	1.6513	1.5828	1.5644
XGB	**1.6518**	1.6582	**1.6093**	0.9991	1.7814	1.7534	1.6788	1.6603	1.5653

From Table 7, we can see that the MDD of S&P 500 index is the lowest and the MDD of BAH is highest in all trading strategies including all machine learning algorithms and BAH strategy in each industry. Through the multiple comparison analysis, we can find that the MDDs of all machine learning algorithms and BAH strategy are significantly greater than that of the S&P 500 index in all industries except PU. Otherwise, there is no significant difference between the MDDs of any two algorithms in the BM, COM, and TECH. In the CC, the MDD of BAH is significantly greater than that of RF and XGB; otherwise, there is no significant difference between the MDDs of any two algorithms. In the EN, the MDD of BAH is significantly greater than that of MLP, DBN, and SAE; otherwise, there is no significant difference between the MDDs of any two algorithms. In the FIN, the MDD of BAH is significantly greater than that of RNN, LSTM, GRU, SVM, XGB, and RF; otherwise, there is no significant difference between the MDDs of any two algorithms. In the IND, the MDD of BAH is significantly greater than that of GRU, CART, LR, NB, SVM, XGB, and RF; otherwise, there is no significant difference between the MDDs of any two algorithms. In the NCC, the MDD of BAH is significantly greater than that of RNN, GRU, CART, NB, RF, and XGB; otherwise, there is no significant difference between the MDDs of any two algorithms. In the PU, there is no significant difference between the MDDs of all trading strategies including BAH strategy and S&P500 index.

**Table 7:** Comparison of MDD of various trading strategies in the different industries of SPICS. Best performance is in boldface.

	BM	CC	COM	EN	FIN	IND	NCC	PU	TECH
Index	**0.1939**	**0.1939**	**0.1939**	**0.1939**	**0.1939**	**0.1939**	**0.1939**	**0.1939**	**0.1939**
BAH	0.4886	0.4525	0.4539	0.7016	0.3929	0.3849	0.3533	0.2726	0.4393
MLP	0.3667	0.3857	0.3843	0.4705	0.3313	0.3436	0.3275	0.2620	0.3952
DBN	0.3680	0.3857	0.3837	0.4730	0.3321	0.3466	0.3282	0.2422	0.3999
SAE	0.3516	0.3751	0.3916	0.4620	0.3328	0.3356	0.3291	0.2500	0.3936
RNN	0.3556	0.3585	0.3760	0.5587	0.3098	0.3149	0.2819	0.2260	0.3764
LSTM	0.4033	0.3622	0.3815	0.5806	0.3109	0.3219	0.2970	0.2254	0.3743
GRU	0.4275	0.3695	0.3893	0.5630	0.3020	0.2984	0.2694	0.2202	0.3588
CART	0.3642	0.3587	0.3549	0.5653	0.3252	0.3094	0.2840	0.2229	0.3703
NB	0.4142	0.3575	0.3487	0.5900	0.3281	0.3028	0.2840	0.2043	0.3661
RF	0.3813	0.3187	0.3532	0.5896	0.3007	0.2863	0.2803	0.2109	0.3588
LR	0.3823	0.3611	0.3830	0.5590	0.3221	0.3014	0.2948	0.2193	0.3761
SVM	0.3862	0.3519	0.3529	0.5793	0.3251	0.2983	0.2973	0.2315	0.3612
XGB	0.3737	0.3421	0.3329	0.5845	0.3146	0.3022	0.2723	0.2110	0.3737

#### Comparative analysis of intelligent trading algorithms for each industry in CSICS

In the CSICS, we still use the analysis method mentioned above. We obtain the best trading algorithm which can be suitable for the stock trading of the given industry by comparing the performance of different algorithms. This can provide some guidance for the formulation of an investment strategy.

We can see from Table 8 that the WR of MLP is the highest in every industry. In the BM, CC, COM, FIN, IND, NCC, PU, and TECH, the WR of MLP is not a significant difference from that of DBN, SAE, and SVM through multiple comparison analysis, but the WRs of MLP, DBN, and SAE are significantly higher than that of other algorithms. In the EN, the WR of MLP is not significantly different from that of DBN, SAE, RNN, SVM, and NB, but the WRs of MLP, DBN, and SAE are significantly higher than that of other algorithms. Therefore, MLP, DBN, and SAE perform well in all industries.

**Table 8:** Comparison of the WR of different trading algorithms in the different industries of CSICS. Best performance is in boldface.

	BM	CC	COM	EN	FIN	IND	NCC	PU	TECH
MLP	**0.7077**	**0.8242**	**0.8211**	**0.7219**	**0.7117**	**0.7653**	**0.7339**	**0.8586**	**0.7975**
DBN	0.7008	0.8090	0.7971	0.7125	0.6993	0.7587	0.7217	0.8398	0.7856
SAE	0.7007	0.8116	0.7978	0.7095	0.6991	0.7594	0.7211	0.8366	0.7899
RNN	0.5394	0.5472	0.5653	0.5330	0.5331	0.5439	0.5290	0.5593	0.5514
LSTM	0.5067	0.5109	0.4971	0.4851	0.5013	0.4981	0.5022	0.4824	0.5122
GRU	0.5063	0.5027	0.4932	0.4991	0.5044	0.5007	0.5047	0.5026	0.5147
CART	0.5034	0.5015	0.5175	0.4929	0.5039	0.4985	0.4869	0.5064	0.5183
NB	0.5185	0.4830	0.5102	0.5398	0.5190	0.5197	0.5000	0.5311	0.5203
RF	0.5061	0.5147	0.5253	0.5187	0.5162	0.5172	0.4996	0.5406	0.5255
LR	0.4949	0.5153	0.5238	0.4954	0.5074	0.5058	0.5094	0.4855	0.5224
SVM	0.5576	0.5706	0.5945	0.5682	0.5674	0.5714	0.5465	0.6194	0.5750
XGB	0.5043	0.5052	0.5241	0.5062	0.5071	0.5104	0.5085	0.5213	0.5219

We can see from Table 9 that the ARRs of the CSI 300 index and BAH strategy are less than that of all other trading algorithms in each industry. The ARR of NB is the highest in all industries except for the TECH, and the ARR of MLP is the highest in the TECH. Through the analysis of variance and multiple comparative analysis, the ARRs of all trading algorithms are significantly higher than that of CSI 300 index and BAH strategy. In all the mentioned machine learning algorithms in this paper, although the ARRs of some algorithms seem very better than that of other algorithms, there is no significant difference between the ARRs of them in all industries except the IND. It is worth noting that the ARR of RF is significantly lower than that of other algorithms in the IND, but there is no significant difference between other algorithms. As far as ARR is concerned, the traditional machine learning algorithms are not worse than that of all the algorithms based on the deep neural network in most industries.

**Table 9:** Comparison of the ARR of various trading strategies in the different industries of CSICS. Best performance is in boldface.

	BM	CC	COM	EN	FIN	IND	NCC	PU	TECH
Index	0.0680	0.0529	0.0530	0.0704	0.0584	0.0625	0.0695	0.0822	0.0660
BAH	0.1201	0.3208	0.2622	0.1107	0.1952	0.2050	0.3113	0.1514	0.3526
MLP	0.4689	0.7658	0.6645	0.4418	0.5489	0.5520	0.6055	0.4500	**0.7023**
DBN	0.4687	0.7660	0.6590	0.4640	0.5456	0.5511	0.5909	0.4544	0.6880
SAE	0.4735	0.7667	0.6718	0.4332	0.5370	0.5534	0.5921	0.4317	0.6916
RNN	0.4222	0.7081	0.6213	0.4501	0.5245	0.4967	0.5145	0.4138	0.6202
LSTM	0.4305	0.7196	0.5513	0.4201	0.4916	0.4926	0.5503	0.3494	0.6507
GRU	0.4408	0.6948	0.5625	0.4353	0.4940	0.4584	0.5670	0.4057	0.6392
CART	0.4524	0.8318	0.6172	0.4506	0.5375	0.4906	0.6256	0.3214	0.6921
NB	**0.4915**	**0.8987**	**0.7072**	**0.4943**	**0.5858**	**0.5627**	**0.7083**	**0.4937**	0.6820
RF	0.3812	0.7165	0.5494	0.4254	0.4888	0.4142	0.5144	0.3889	0.5840
LR	0.4266	0.6839	0.5917	0.4414	0.4974	0.4568	0.5637	0.3853	0.6168
SVM	0.3931	0.6941	0.5962	0.4227	0.5052	0.4539	0.5352	0.4000	0.5732
XGB	0.4038	0.6856	0.5635	0.3937	0.4855	0.4496	0.5410	0.4162	0.5785

We can see from Table 10, the ASRs of CSI 300 index and BAH strategy are lower than that of all machine learning algorithms. In BM, COM, FIN, and NCC, the ASR of LR is the highest; in CC, IND, and TECH, the ASR of LSTM is the highest; in EN and PU, the ASR of GRU is the highest. Through the analysis of variance and multiple comparative analysis, the ASRs of CSI 300 index and BAH strategy are significantly lower than that of all other machine learning algorithms. In the CC, EN, PU, TECH, there is no significant difference between the ASRs of all algorithms. In the BM, the ASR of NB is significantly lower than that of LR and GRU, otherwise, there is no significant difference between any two algorithms. In the COM, the ASR of NB is significantly lower than that of LR, otherwise, there is no significant difference between any two algorithms. In the FIN, the ASR of NB is significantly lower than that of all other algorithms; the ASR of CART is significantly lower than that of all other algorithms except MLP, DBN, and SAE; otherwise, there is no significant difference between any two algorithms. In the IND, the ASR of NB is significantly lower than that of all other algorithms; the ASR of CART is significantly lower than that of LSTM; otherwise, there is no significant difference between any two algorithms. In the NCC, the ASR of CART is significantly lower than that of LR; otherwise, there is no significant difference between any two algorithms. As far as ASR is concerned, the NB and CART are not the ideal choices. It is worth noting that the traditional machine learning algorithms are not worse than the popular algorithms based on the deep neural network in some industries.

**Table 10:** Comparison of the ASR of various trading strategies in the different industries of CSICS. Best performance is in boldface.

	BM	CC	COM	EN	FIN	IND	NCC	PU	TECH
Index	0.2818	0.2148	0.2254	0.2896	0.2435	0.2591	0.2891	0.3495	0.2681
BAH	0.2644	0.5879	0.5251	0.2186	0.3874	0.4310	0.7450	0.3679	0.6902
MLP	1.2389	1.6380	1.4765	1.1380	1.3693	1.3372	1.6794	1.2399	1.5452
DBN	1.2376	1.6491	1.4726	1.2017	1.3648	1.3358	1.6451	1.2561	1.5192
SAE	1.2514	1.6523	1.5072	1.1244	1.3427	1.3405	1.6536	1.1887	1.5215
RNN	1.2705	1.7502	1.6657	1.3334	1.4679	1.4194	1.6563	1.3651	1.6173
LSTM	1.3661	**1.8788**	1.5563	1.3215	1.4317	**1.4834**	1.8393	1.2527	**1.8263**
GRU	1.3896	1.8132	1.5360	**1.4054**	1.5016	1.4168	1.8716	**1.4398**	1.7625
CART	1.0845	1.4502	1.3141	1.0355	1.1687	1.1433	1.5246	0.8780	1.4251
NB	0.9071	1.3242	1.1568	0.8363	1.0233	0.9924	1.6110	0.8037	1.4287
RF	1.2148	1.8485	1.5106	1.3246	1.4367	1.2530	1.7142	1.3439	1.5838
LR	**1.3950**	1.7961	**1.6896**	1.3905	**1.5233**	1.4040	**1.9249**	1.4087	1.7161
SVM	1.2003	1.7266	1.5861	1.2429	1.4184	1.3082	1.7124	1.3122	1.4883
XGB	1.2993	1.7767	1.5741	1.2124	1.4216	1.3590	1.7972	1.4158	1.5807

We can see from the Table 11, the MDD of CSI 300 index is lower than that of all machine learning algorithms and BAH strategy in all industries except the CC and NCC; in the CC, the MDD of RF is the lowest; in the NCC, the MDD of LR is the lowest. Through analysis of variance and multiple comparative analysis, the MDD of the CSI 300 index is not significantly lower than that of LR and GRU in the BM, but significantly lower than that of other algorithms and BAH strategy; the MDD of the BAH strategy is significantly higher than that of LR and GRU, but there is no significant difference between BAH and other algorithms; the MDD of NB is significantly higher than that of LSTM, GRU, CART, LR, and XGB; otherwise, there is no significant difference between any two algorithms. In the CC, the MDD of the CSI 300 index is significantly lower than that of NB and BAH strategy, but there is no significant difference between CSI 300 index and other algorithms; the MDD of the BAH strategy is significantly higher than that of RNN, LSTM, GRU, and RF, but there is no significant difference between BAH strategy and other algorithms; the MDD of the NB is significantly higher than that of RNN, LSTM, GRU, CART, SVM, RF, and XGB; otherwise there is no significant difference between any two algorithms. In the COM, the MDD of the CSI 300 index is significantly lower than that of NB and BAH strategy, but there is no significant difference between CSI 300 index and other algorithms; there is no significant difference between BAH and other algorithms; the MDD of the NB is significantly higher than that of LR and XGB; otherwise, there is no significant difference between any two algorithms. In the EN, the MDD of the CSI 300 index is significantly lower than that of NB and BAH, but there is no significant difference between CSI 300 index and other algorithms; there is no significant difference between BAH and other algorithms; the MDD of NB is significantly higher than that of LSTM, GRU, RF, and LR; otherwise, there is no significant difference between any two algorithms. In the FIN, the MDD of the CSI 300 index is significantly lower than that of MLP, DBN, SAE, NB, and BAH, but there is no significant difference between CSI 300 index and other algorithms; the MDD of the BAH strategy is significantly higher than that of RNN, LSTM, GRU, CART, LR, SVM, and RF, but there is no significant difference between BAH and other algorithms; the MDD of the NB is significantly higher than that of all other algorithms; otherwise, there is no significant difference between any two algorithms. In the IND, the MDD of the CSI 300 index is significantly lower than that of all other algorithms and BAH strategy; the MDD of the BAH strategy is significantly higher than that of RNN, LSTM, GRU, CART, LR, and XGB, but there is no significant difference between BAH strategy and other algorithms; the MDD of the NB is significantly higher than that of all other algorithms; otherwise, there is no significant difference between any two algorithms. In the NCC, there is no significant difference between the MDDs of CSI 300 index, BAH strategy, and other algorithms. In the PU, there is no significant difference between the MDDs of CSI 300 index, BAH and other algorithms except NB, and the MDD of the NB is significantly higher than that of CSI 300 index, BAH, and other algorithms; otherwise, there is no significant difference between any two algorithms. In the TECH, the MDD of the CSI 300 index is significantly lower than that of MLP, DBN, SAE, NB and BAH strategy, but there is no significant difference between CSI 300 index and other algorithms; the MDD of the DBN is significantly higher than that of LSTM; otherwise there is no significant difference between any two algorithms.

**Table 11:** Comparison of the MDD of various trading strategies in the different industries of CSICS. Best performance is in boldface.

	BM	CC	COM	EN	FIN	IND	NCC	PU	TECH
Index	**0.4750**	0.5022	**0.4873**	**0.4713**	**0.4786**	**0.4822**	0.4680	**0.4568**	**0.4998**
BAH	0.7327	0.7026	0.6840	0.7199	0.6399	0.6982	0.5623	0.6459	0.6667
MLP	0.6591	0.6153	0.6406	0.6334	0.5671	0.6402	0.5281	0.5752	0.6397
DBN	0.6529	0.6092	0.6196	0.6135	0.5673	0.6432	0.5354	0.5988	0.6517
SAE	0.6417	0.6165	0.6352	0.6334	0.5744	0.6439	0.5445	0.6196	0.6468
RNN	0.6544	0.5268	0.5698	0.5937	0.5293	0.5913	0.5199	0.5169	0.5678
LSTM	0.6149	0.5043	0.6533	0.5570	0.5404	0.5588	0.4735	0.5046	0.5165
GRU	0.5846	0.5245	0.5737	0.5341	0.5314	0.5888	0.4574	0.4929	0.5342
CART	0.6067	0.5639	0.5860	0.6072	0.5409	0.6042	0.4778	0.5659	0.5872
NB	0.8009	0.7675	0.7736	0.8133	0.7227	0.8019	0.5958	0.8052	0.6811
RF	0.6422	**0.4884**	0.5514	0.5691	0.5343	0.6376	0.4972	0.4823	0.6027
LR	0.5946	0.5829	0.5250	0.5541	0.4973	0.5891	**0.4544**	0.4793	0.5619
SVM	0.6628	0.5444	0.5996	0.6005	0.5435	0.6104	0.4894	0.5039	0.6148
XGB	0.6068	0.5611	0.5279	0.6293	0.5557	0.5873	0.4704	0.4870	0.5874

### Selection of the optimal trading model for different industries

Next, we give the optimal trading algorithms (TOTAs) for stock trading of each industry based on the analysis results of the above. We give a series of rules as follows, where “*a*>*b*” represents that the performance of algorithm *a* is significantly greater than that of algorithm *b*; “*a* = *b*” represents that the performance of algorithm *a* is no significantly different from that of algorithm *b*. (*a* = *b*)∧(*b*>*d*) represents that “*a* = *b*”and “*b*>*d*”are simultaneously established.

For any industry *i*∈{*BM*,*CC*,*COM*,*EN*,*FIN*,*IND*,*NCC*,*PU*,*TECH*}, for any performance evaluation indicator *j*∈{*WR*,*ARR*,*ASR*,−*MDD*}. We all know that the greater the value of *WR*,*ARR*,*ASR* and −*MDD*, the better the trading performance of the strategy or trading models. The relationship of the performance of all strategies including machine learning algorithms, BAH strategy, and benchmark index can be expressed by the relationship among the 3 strategies, which are expressed as *a*,*b*, and *c* respectively.

Rule 1. Sifting Rule Depending on Single Indicator

- If (*a*>*b*)∧(*b*>*c*)∧(*a*>*c*), then the strategy *a* is the optimal in all strategies;
- If (*a*>*b*)∧(*b*>*c*)∧(*a* = *c*), then the strategy *a* is the optimal in all strategies;
- If (*a*>*b*)∧(*b* = *c*)∧(*a* = *c*), then the strategy *a* is the optimal in all strategies;
- If (*a*>*b*)∧(*b* = *c*)∧(*a*>*c*), then the strategy *a* is the optimal in all strategies;
- If (*a* = *b*)∧(*b*>*c*)∧(*a*>*c*), then the strategy *a* and *b* are the optimal in all strategies;
- If (*a* = *b*)∧(*b* = *c*)∧(*a* = *c*), then the strategy *a*,*b*, and *c* are the optimal in all strategies.

Firstly, we want to choose the optimal machine learning algorithms for each industry in which the trading performance of the algorithm can be significantly better than that of the benchmark index, that is, algorithm trading strategy can beat the market; secondly, the trading performance of the optimal machine learning algorithm can be significantly better than the BAH strategy in each industry, which is conducive to take an active quantitative investment strategy for stock trading while reducing risk. Therefore, if the trading performance of machine learning algorithms is not better than that of the index, we hope that it is significantly better than BAH strategy. Otherwise, the machine learning algorithm will not make sense for stock trading. We select the optimal trading algorithms(TOTAs) which are significantly better than the rest of the algorithms, as shown in Table 12.

**Table 12:** TOTAs are selected according to a single indicator.

Industry	Indicator	SPICS	CSICS
BM	WR	MLP, DBN, SAE	MLP, DBN, SAE
	ARR	Any trading algorithm can be used (ATAU).	ATAU
	ASR	ATAU	GRU, LR
	MDD	ATAU	GRU, LR
CC	WR	MLP, DBN, SAE	MLP, DBN, SAE
	ARR	ATAU	ATAU
	ASR	LR	ATAU
	MDD	RF, XGB	RNN, LSTM, GRU, RF
COM	WR	MLP	MLP, DBN, SAE
	ARR	ATAU	ATAU
	ASR	ATAU	LR
	MDD	ATAU	LR, XGB
EN	WR	ATAU	MLP, DBN, SAE
	ARR	ATAU	ATAU
	ASR	SAE	ATAU
	MDD	MLP, DBN, SAE	LSTM, GRU, RF, LR
FIN	WR	MLP, DBN, SAE	MLP, DBN, SAE
	ARR	MLP	LSTM, RNN, GRU, SVM, LR, RF, XGB
	ASR	Any trading algorithm can be used except CART.	MLP, DBN, SAE, RNN, LSTM, GRU
	MDD	RNN, LSTM, GRU, SVM, XGB, RF	RNN, LSTM, GRU, CART, LR, SVM, RF
IND	WR	MLP, DBN, SAE	MLP, DBN, SAE
	ARR	ATAU	LSTM
	ASR	RF	MLP, DBN, SAE, RNN, LSTM, GRU
	MDD	GRU, CART, RF, LR, SVM, NB, XGB	RNN, LSTM, GRU, CART, LR, XGB
NCC	WR	MLP, DBN, SAE	MLP, DBN, SAE
	ARR	ATAU	ATAU
	ASR	RF	LR
	MDD	RNN, GRU, CART, NB, RF, XGB	ATAU
PU	WR	MLP, DBN, SAE	MLP, DBN, SAE
	ARR	ATAU	ATAU
	ASR	ATAU	ATAU
	MDD	ATAU	Any trading algorithm can be used except for NB.
TECH	WR	MLP, DBN, SAE	MLP, DBN, SAE
	ARR	ATAU	ATAU
	ASR	ATAU	ATAU
	MDD	ATAU	LSTM

From Table 12, we find that the optimal trading model based on the WR is always found in any industry, and MLP is the optimal algorithms in all industries through the analysis of the industries in the SPICS; MLP is the optimal trading model based on ARR in the FIN, and any algorithms can be used in other industries; the optimal trading model based on ASR can be found in the CC, EN, FIN, IND, and NCC, and any algorithms can be used in other industries; the optimal trading model based on MDD can be found in the CC, EN, FIN, IND, and NCC, and any algorithms can be used in other industries. Through the analysis of the industries in the CSICS, the optimal model can be found in all industries based on the WR, and SAE, MLP, and DBN are the optimal trading models in most industries; TOTAs based on ARR can be found in the FIN and IND, and any algorithms can be used in other industries; TOTAs based on ASR can be found in the BM, COM, FIN, IND, and NCC, and any algorithms can be used in other industries; TOTAs based on MDD can be found in all industries except NCC. From Table 12, we can find that there is more than one optimal trading algorithm in some industries, which is normal. In fact, there is no significant difference in performance among the multiple optimal trading algorithms selected. For example, for the industry BM in SPICS, we obtain the optimal trading algorithms which including MLP, DBN, and SAE based on WR. These three algorithms have no statistically significant difference for WR.

However, we can see from Table 12 that there are too many “ATAUs”, which means that the optimal trading models proposed for each industry are still not refined enough, so we propose a new set of rules based on Table 12 to narrow the selection range of the optimal models. For each industry, ASR represents risk-adjusted returns, it is the most important indicator for evaluating a trading algorithm; secondly, ARR represents the return of a stock during a holding period, so ARR is also an important indicator for evaluating the trading algorithm without considering risk; thirdly, MDD describes the potential risks of trading algorithms which are applied to stock trading; finally, WR represents the performance of a trading algorithm in predicting stock price trends, which is not a direct source of stock investment returns. Therefore, we assume that *ASR*≻*ARR*≻*MDD*≻*WR* according to the importance of the four evaluation indicators, where “*m*≻*n*” represents the indicator *m* is more important than the indicator *n*. The following refining rules are proposed.

Rule 2. Refining Rules Depending on Comprehensive Indicators

**If** *ASR*∩*ARR*∩*MDD*∩*WR*≠∅, **then** TOTAs = *ASR*∩*ARR*∩*MDD*∩*WR*;

    **else if** *ASR*∩*ARR*∩*MDD*∩*WR*≠∅ **and** *ASR*∩*ARR*∩*MDD*≠∅, **then** TOTAs = *ASR*∩*ARR*∩*MDD*;

        **else if** *ASR*∩*ARR*∩*MDD*≠∅ **and** *ASR*∩*ARR*≠∅, **then** TOTAs = *ASR*∩*ARR*;

            **else** *ASR*∩*ARR*≠∅, **then** TOTAs = *ASR*.

For example, we can use the above rules to select TOTAs for NCC in SPICS: *WR* = {*MLP*,*DBN*,*SAE*}, *ARR* = *ATAU* = {*MLP*,*DBN*,*SAE*,*RNN*,*GRU*,*LSTM*,*NB*,*SVM*,*XGB*,*LR*,*RF*,*CART*}, *ASR* = {*RF*}, *MDD* = {*RNN*,*GRU*,*CART*,*NB*,*RF*,*XGB*}, we have *ASR*∩*ARR*∩*MDD*∩*WR*≠∅ and *ASR*∩*ARR*∩*MDD* = *RF*≠∅, so the RF is the TOTA for NCC in SPICS. We obtain TOTAs for each industry in the SPICS and CSICS, as shown in Table 13.

**Table 13:** TOTAs are selected according to the refining rules.

Industry	SPICS	CSICS
BM	MLP, DBN, SAE	GRU, LR
CC	LR	RNN, LSTM, GRU, RF
COM	MLP	LR
EN	SAE	LSTM, GRU, RF, LR
FIN	MLP	LSTM, RNN, GRU
IND	RF	LSTM
NCC	RF	LR
PU	MLP, DBN, SAE	MLP, DBN, SAE
TECH	MLP, DBN, SAE	LSTM

As can be seen from Table 13, the number of optimal trading models selected according to Rule 2 is small because Rule 2 takes into account the importance between the ASR and the remaining indicators. The transaction models selected are more operational. At the same time, deep neural network algorithms have a good performance in most industries, but LR and RF are very prominent in some industries.

These experimental results show that on SPICS and CSICS, we can always select TOTAs based on the single indicator and comprehensive indicators in all industries. We can apply TOTAs to implement trading activity in each industry of China A-share market and American market.

## Conclusion

In this paper, we adopt the 424 SPICS in the US market and the 185 CSICS in China market from 9 industries as the research object. Then, for each stock in every industry, we select the data of the 2000 trading days before December 31, 2017, and build 44 technical indicators as the input features of the machine learning algorithm to predict the trends of the stock price. Then, we formulate trading strategies based on the trading signals, analyze and evaluate the performance of these algorithms in different industries. Finally, we use a set of rules to select TOTAs for stock trading in each industry. The experiment shows that on SPICS and CSICCS, we can select at least one of the best trading models for each industry based on the single indicator and comprehensive indicators. The optimal trading models perform well for WR in all industries; the ARR and ASR of the model can be significantly better than that of the benchmark index and BAH strategy in most industries; the MDD of the model can be significantly less than that of BAH strategy in most industries. Therefore, the algorithms can be applied to the stock investment in most industries, and it is a very significant effect on investment yield and risk management.

In view of the rapid development of artificial intelligence technology and the easy access to financial big data, the future research work can be carried out from the following aspects: (1) using the deep neural network to carry out dynamic portfolio management among different assets; (2) using the deep neural network to simulate high-frequency trading and develop strategies. The solution for these problems will help to develop an advanced and profitable automated trading system based on financial big data, which including dynamic portfolio construction, optimal execution, and risk management according to the changes in market conditions.

## Supporting information

S1 Table Features used in the machine learning algorithms. (PDF)

S2 Table The stock symbols for different industries in both SPICS and CSICS. (PDF)
