# Supplementary material for: Selection of the optimal trading model for stock investment in different industries
Source: PLoS One. 2019 Feb 13;14(2):e0212137. doi: 10.1371/journal.pone.0212137 (PMC6373956; doi:10.1371/journal.pone.0212137)
Supplement: S1 Table — (PDF) [file pone.0212137.s001.pdf]

S1 Table. Features used in the Machine Learning algorithms

1.Basic symbols	Explanation
H[i]	H[i] represents the highest price of a stock on the i day, where H indicates the highest price time series of a stock.
L[i]	L[i] represents the lowest price of a stock on the i day, where H indicates the lowest price time series of a stock.
C[i]	C[i] represents the closing price of a stock on the i day, where H indicates the closing price time series of a stock.
O[i]	O[i] represents the opening price of a stock on the i day, where H indicates the opening price time series of a stock.
V[i]	V[i] represents the volume of a stock on the i day, where H indicates the volume time series of a stock.
SMA(x, n)	The n order simple moving average of the time series x.
EMA(x, n)	The n order exponentially moving average of the time series x.
1：N	1:N represents all positive integers from 1 to N.
runSum(x, n)	runSum(x, n) indicates the rolling sum of the order n of the sequence x, for example, x=1,2,3,4,5,6,7, then runSum (x, 3) is NA, NA, 6, 9, 12, 15, 18.
HH[i]	HH[i] represents the maximum value in the highest price sequence.
LL[i]	LL[i] represents the minimum value in the lowest price sequence.
runMean(x, n)	runMean(x, n) represents the rolling mean of the n order of the sequence x.
runSD(x, n)	runSD(x, n) represents the rolling standard deviation of the n order of the sequence x.

2.Features	Calculation method	Explanation
(1) ATR	TR[i]=max(H[i]-C[i], |C[i-1]-H[i]|, |C[i-1]-L[i]|)，<br>ATR=SMA(TR, 14)	The ATR is a Welles Wilder style moving average of the True Range. The ATR is a measure of volatility. High ATR values indicate high volatility, and low values indicate low volatility.
(2) ADX	http://www.fmlabs.com/reference/default.htm?url=DI.htm<br>http://www.fmlabs.com/reference/default.htm?url=DX.htm<br>http://www.fmlabs.com/reference/default.htm?url=ADX.htm	The ADX is a Welles Wilder style moving average of the Directional Movement Index (DX). The values range from 0 to 100, but rarely get above 60. To interpret the ADX, consider a high number to be a strong trend, and a low number, a weak trend.
(3) OBV	If C[i]>C[i-1]，OBV[i]=OBV[i]+V[i]<br>If C[i]<C[i-1]，OBV[i]=OBV[i]-V[i]<br>If C[i]=C[i-1]，OBV[i]=OBV[i]	The On Balance Volume (OBV) is a cumulative total of the up and down volume. A series of rising peaks, or falling troughs, in the OBV indicates a strong trend. If the OBV is flat, then the market is not trending.
(4) WR	WR[n]=100*（HH[1:n]-C[n]）/(HH[1:n]-LL[1:n])	The values range from zero to 100 and are charted on an inverted scale, that is, with zero at the top and 100 at the bottom. Values below 20 indicate an overbought condition and a sell signal is generated when it crosses the 20 line. Values over 80 indicate an oversold condition and a buy signal is generated when it crosses the 80 line.
(5)RSI	If C[i]>C[i-1], then up[i]=C[i]-C[i-1], dn[i]=0;<br>If C[i]<=C[i-1], then dn[i]=C[i-1]-C[i], up[i]=0;<br>Upave[i]=(upave*(i-1)+up)/(i);<br>Dnave[i]=(dnave*(i-1)+dn)/(i);<br>RSI[i]=100*upave[i]/(upave[i]+dnave[i])	The RSI is interpreted as an overbought/oversold indicator when the value is over 70/below 30. You can also look for divergence with price. If the price is making new highs/lows, and the RSI is not, it indicates a reversal.
(6) CMF	VA=(C-(H+L)/2)/(H-L)*V<br>CMF[i]=runSum(VA, i)/runSum(V, i)	When the Chaikin Money Flow(CMF) is above 0.25 it is a bullish signal, when it is below -0.25, it is a bearish signal. If the CMF remains below zero while the price is rising, it indicates a probable reversal.
(7) BandPer	PB=(C-BBDN)/(BBUP-BBDN),where BBDN is the lower track value of the Bollinger bands, and BBUP is the lower track value of the Bollinger bands.	BandPer index can tell us where the current price is in the Bollinger line, which can be used for morphological identification and quantitative trading.
(8) BandWid	BW=(BBUP-BBDN)/BBMA, where BBMA is the value of the middle track of the Bollinger bands.	BandWid is a measure of volatility. The BandWid value is higher when volatility is high, and lower when volatility is low.
(9) Chaikin A/D Oscillator	AD[i]=AD[i-1]+(C[i]-L[i])+(((C[i]-L[i])-(H[i]-C[i]))/(H[i]-L[i]+0.01))*V[i];<br>CO=EMA(AD,3）- EMA(AD,10）.	Chaikin A/D Oscillator is a stock index related to trading volume, which can be used to observe the flow of funds in the market.
(10) DIS	DIS=C/SMA(C, 20))*100	Disparity Index can measure the relative position of the most recent closing price to a selected moving average and reports the value as a percentage.
(11) EOM	EOM[1]=(H[1]+L[1])/2;<br>EOM[i]=((H[i]+L[i])/2-(H[i-1]+L[i-1])/2)*(H[i]-L[i])/V[i].	Ease of Movement Value Index is used to relate an asset's price change to its volume. Ease of Movement highlights the relationship between volume and price changes and is particularly useful for assessing the strength of a trend
(12) FI	FI[i]=(C[i]-C[i-1])*V[i];<br>FI=SMA(FI, 2).	The force index (FI) is used to illustrate how strong the actual buying or selling pressure is. High positive values mean there is a strong rising trend, and low values signify a strong downward trend.
(13) MAO	MAO=SMA(C, 12)-SMA(C, 26).	MA oscillator index is the difference of the moving average of two different time periods, reflecting the degree of swinging of stock prices.
(14) MFI	http://www.fmlabs.com/reference/default.htm?url=MoneyFlowIndex.htm	The Money Flow Index calculates the ratio of money flowing into and out of a security

(15) MI	r=H-L;<br>ema1=EMA(r, 9);<br>ema2=(EMA(r, 9))^2;<br>x=ema1/ema2;<br>MI=runSum(x, 9).	Mass Index Momentum is used to predict trend reversals. It is based on the notion that there is a tendency for reversal when the price range widens, and therefore compares previous trading ranges (highs minus lows).
(16) MOM	MOM[i]=(C[i]/C[i-9])*100.	Momentum Index is the (rate of)<br>change of a series over n periods.
(17) NCO	NCO[i]=C[i]-C[12].	Net Change Oscillator Index is the change of series over n periods.
(18) PO	PO=(SMA(C, 5)-SMA(C, 10))/(SMA(C, 10)).	Price Oscillator Index removes the trend in prices by subtracting a moving average of the price from the price. The PO shows cycles and overbought/oversold conditions.
(19) PSY	ROC[i]=(C[i]-C[i-10])/C[i-10];<br>PSY[i]=sum(ROC((i-10):i)>=0)/10.	PSY index reflects the psychological fluctuations of investors in the stock market.
(20) RMI	mo[i]=C[i]-C[i-1];<br>RMI[i]=sum(mo[mo[(i-13):i]>=0])/(sum(mo[mo[(i-13):i]>=0])+sum(mo[mo[(i13):i]<0])+0.01);<br>where mo[mo>=0] represents a sequence consisting of values greater than 0 in sequence mo. sum (x) represents the sum of all the values in the sequence x.	Relative Momentum Index is a swinging indicator, which shows the same strength and weakness as other overbought / oversold indicators.
(21) ROC	ROC[i]=log(C[i]/C[i-1]).	The ROC indicator provides the percentage difference of a series over two observations
(22) SROC	SROC=(EMA(C, 20)/EMA(C,10))*100.	Smoothed Rate of Change Index, like ROC, is used to reflect the rate of change in stock prices.
(23) SONAR	SONAR=MOM(EMA(C, 25), 25).	Sonar index is the (rate of) change of exponential moving mean of the closing price over n periods.
(24) SONSIG	SONSIG=EMA(SONAR, 9).	SONSIG index is exponential moving mean of SONAR series over n periods.
(25) TRIX	M=EMA(EMA(EMA(C,20),20),20);<br>TRIX[i]=100*(M[i]-M[i-1])/M[i].	The TRIX indicator calculates the rate of change of a triple exponential moving average.
(26) VMA	VMA=SMA(VO, n).	Moving Average of the Volume
(27) VOS	Vm=EMA (V, 12);<br>Vn=EMA (V, 26);<br>VOS=((Vm-Vn)/Vn)*100.	Volume Oscillator index can analyze the trend of turnover and judge the direction of trend change in time.
(28) VROC	VROC[i]= log(V[i]/V[i-13]).	VROC index applies movement of the volume of to measure the trend of volume turnover in order to detect the strength of supply and demand in advance.
(29) Return	Ret=log(C[i]/C[i-1]);<br>Return=runMean(Ret, 14).	Return represents means of logarithmic return rate over a n-period moving window.
(30) Sigma	Ret=log(C[i]/C[i-1]);<br>Return=runSD(Ret, 14).	Sigma represents standard deviations of logarithmic return rate over a n-period moving window.
(31) CCI	TP[i]=(HH[1:i]+LL[i]+C[i])/3;<br>ATP=SMA(TP, 20);<br>MDTP=runMean(|TP-ATP|,20);<br>CCI=(TP-ATP)/(0.015*MDTP).	The Commodity Channel Index (CCI) attempts to identify starting and ending trends.
(32) RSV	RSV[i]=100*（C[i]-LL[(i-8):i]）/(HH[(i-8):i]-LL[(i-8):i]).	The RSV index is mainly used to analyze whether the market is in an overbought or oversold state. The market is overbought when RSV is higher than 80%; The market was oversold when RSV was below 20%.
(33) Kvalue	Kvalue=EMA(RSV,2)	The K, D, and J index can be used to judge the market more quickly and intuitively and is widely used in the analysis of the short and medium term trend of the stock market.
(34) Dvalue	Dvalue=EMA(Kvalue,2)	
(35) Jvalue	Jvalue=3*Kvalue-2*Dvalue	
(36) MACD	Fast=EMA(C,12）<br>Slow=EMA (C, 26)<br>DIF=Fast-Slow<br>MACD=EMA(DIF, 9)	The MACD signals trend changes and indicates the start of new trend direction.
(37) CAD	CAD[i] =CAD[i-1] + V[i]* CLV[i]<br>其中 CLV [i]= (2*C[i]–H[i]–L[i]) / (H[i]–L[i])	The Chaikin Accumulation / Distribution (CAD) line is a measure of the money flowing into or out of a security. It is similar to OBV.
(38) VOLA	EMAHL=EMA(H-L,10)<br>VOLA[i]=(EMAHL[i]-EMAHL[i-9])/EMAHL[i-9]	Chaikin Volatility measures the rate of change of the security's trading range.
(39) NBIAS	NBIAS=100*(C-SMA(C, 6))/SMA(C, 6)	The NBIAS index reflects the deviation between price and moving average in a certain period and contribute to obtaining the possibility of return or rebound caused by the deviation from the moving average trend in the violent fluctuation.
(40) Ret	Ret[i]=log(C[i]/C[i-1])	Ret represents logarithmic return rate on i day
(41) SMA_5	SMA_5=SMA(C, 5)	SMA_5 represents the arithmetic mean of the closing price over the past 5 days.
(42) SMA_10	SMA_10=SMA(C, 10)	SMA_10 represents the arithmetic moving mean of the closing

		price over the past 10 days.
(43) EMA_5	EMA_5=EMA(C, 5)	EMA_5 represents the exponential moving mean of the closing price over the past 5 days.
(44) EMA_10	EMA_10=EMA(C,10)	EMA_10 represents The exponential moving mean of the closing price over the past 10 days.<br>.
(45) Label	If log(C[i+1]/C[i])>0, Label[i]=1, else Label[i]=0.	The classified label is an important sign to supervise learning algorithm.
