# Supplementary material for: Selection of the optimal trading model for stock investment in different industries
Source: PLoS One. 2019 Feb 13;14(2):e0212137. doi: 10.1371/journal.pone.0212137 (PMC6373956; doi:10.1371/journal.pone.0212137)
Supplement: S2 Table — (PDF) [file pone.0212137.s002.pdf]

S2 Table. The stock symbols for different industries in both SPICS and CSICS.

Dataset	Industry	Stock Symbols
SPICS	BM	EMN IP MON ECL IFF PPG SHW APD VMC PX MOS NUE NEM ATI FMC CF AA FCX
	CC	MAT NWL SBUX GT NKE JCI VFC CMG GPC WMT TIF COST AN MCD DRI RL YUM CCL HAS FAST AZO F MHK CTAS TJX LOW WYNN GPS GWW HOG PHM WHR KSS BBY GME LUV TGT LUK JWN PCAR M WYN KMX CVS HD DAL BWA DHI ROST ORLY FOSL LB MAR DLTR LEN BBBY TSCO
	COM	MSI AMZN NFLX TWX FFIV SNI HSY FOXA CCI JNPR HRS CTL SYMC GLW DIS OMC CSCO CMCSA EXPE IPG PCLN VZ WIN T FTR VIAB EBAY GCI GOOG DISCK DISCA CBS
	EN	VLO FTI XEC SLB EQT RRC COP WMB EOG OXY OKE APC CHK HP SWN APA PXD HAL NOV CNX MRO RDC XOM DVN NFX NBL HES CVX MUR NBR ESV RIG NE DO DNR
	FIN	XL HCP HCN PLD BXP CME AVB VTR VNO PSA MMC AIV AON EQR PGR PNC KEY ALL ESS AFL MET PBCT USB CINF BBT UNM ICE AIZ AMT NDAQ HIG TRV GGP CB RF L ZION FITB HBAN BAC AIG MTB GNW SPG NTRS MAC TROW BK DFS AMP CMA WFC JPM HST AMG C IVZ STT PRU STI TMK BEN ETFC SCHW BLK COF PFG KIM AXP LNC MS CBG WY GS LM
	IND	ROP DHR CHRW GRMN BMS RSG AME MMM SNA SRCL COL PNR LEG TXT LMT IR PH EXPD WM TEL GD ITW LLL APH DE SWK NOC HON RTN DOV TMO A FLS BA WAT FDX UNP BLL NSC CSX GE KSU PKI UPS CMI ROK MAS UTX FLR EMR FLIR JBL CAT MLM OI ETN R COG JEC
	NCC	EW BMY AMGN REGN EL CI DGX CELG PFE MDT PRGO BIIB PAYX MO ABT MDLZ BSX XRAY DPS HUM CL PDCO HRL AET SYY PEP MA UNH KMB LLY KO ADP SYK ALXN PM UHS JNJ TSN TSS BDX PG MNST VAR LH WU BAX PVH ESRX ISRG EFX MRK STZ MYL AVY CAH CAG ADM DVA HRB CCE IRM ADS KR TAP MCO URBN AVP ABC GIS AGN URI MKC V VRTX RHI THC K MCK CPB SJM PWR CLX
	PU	SCG AEP PPL EIX NEE D EXC DUK SO AEE CNP XEL SRE DTE NRG PNW WEC ED NI PEG ETR CMS FE SEE PCG AES
	TECH	QCOM CA FIS MCHP INTU TDC AVGO FSLR CRM FISV ADBE VRSN DNB RHT IBM TXN ACN CTSH XLNX MSFT AKAM WDC ADI CERN EA NVDA ORCL NTAP INTC ADSK XRX HPQ MU KLAC AAPL AMAT STX PBI GOOGL LRCX
CSICS	BM	600010.SS 600019.SS 600111.SS 600219.SS 600309.SS 600352.SS 600362.SS 600489.SS 600547.SS 600549.SS 600585.SS 601600.SS 601899.SS 601958.SS 000060.SZ 000630.SZ 000709.SZ 000792.SZ 000898.SZ 000959.SZ
	CC	600066.SS 600104.SS 600177.SS 600297.SS 600660.SS 600682.SS 600690.SS 600741.SS 600827.SS 000069.SZ 000100.SZ 000559.SZ 000625.SZ 002024.SZ
	COM	600050.SS 600373.SS 600485.SS 600498.SS 600522.SS 600637.SS 000063.SZ 000839.SZ 002027.SZ
	EN	600028.SS 600157.SS 600188.SS 600583.SS 600688.SS 600871.SS 601088.SS 601857.SS 601898.SS 000723.SZ 000983.SZ
	FIN	600000.SS 600015.SS 600016.SS 600030.SS 600036.SS 600048.SS 600061.SS 600109.SS 600208.SS 600369.SS 600376.SS 600383.SS 600390.SS 600606.SS 600649.SS 600663.SS 600816.SS 600837.SS 600895.SS 601009.SS 601099.SS 601166.SS 601169.SS 601318.SS 601328.SS 601398.SS 601601.SS 601628.SS 601939.SS 601988.SS 601998.SS 000001.SZ 000002.SZ 000402.SZ 000540.SZ 000627.SZ 000671.SZ 000686.SZ 000728.SZ 000750.SZ 000783.SZ 000961.SZ 002142.SZ 002146.SZ
	IND	600009.SS 600018.SS 600029.SS 600031.SS 600038.SS 600068.SS 600089.SS 600115.SS 600118.SS 600153.SS 600170.SS 600221.SS 600233.SS 600372.SS 600406.SS 600415.SS 600482.SS 600685.SS 600704.SS 600739.SS 600820.SS 600893.SS 601006.SS 601111.SS 601186.SS 601333.SS 601390.SS 601766.SS 601866.SS 601872.SS 601919.SS 000008.SZ 000157.SZ 000338.SZ 000415.SZ 000425.SZ

		000738.SZ 000768.SZ 000826.SZ 002074.SZ 002081.SZ 002202.SZ
	NCC	600085.SS 600196.SS 600276.SS 600332.SS 600436.SS 600518.SS 600519.SS 600535.SS 600887.SS 000423.SZ 000538.SZ 000568.SZ 000623.SZ 000858.SZ 000876.SZ 000895.SZ 000963.SZ 002007.SZ 002044.SZ
	PU	600008.SS 600011.SS 600021.SS 600674.SS 600795.SS 600886.SS 600900.SS 601991.SS
	TECH	600074.SS 600100.SS 600271.SS 600570.SS 600588.SS 600703.SS 600804.SS 000413.SZ 000503.SZ 000725.SZ 000938.SZ 002008.SZ 002065.SZ 002153.SZ 002174.SZ 002230.SZ 002236.SZ 002241.SZ
